# Supplementary material for: Mantle Cell Lymphoma Presenting as Acute Abdominal Syndrome: A Rare Case Report and Literature Review
Source: Healthcare (Basel). 2021 Aug 5;9(8):1000. doi: 10.3390/healthcare9081000 (PMC8391909; doi:10.3390/healthcare9081000)
Supplement: Supplementary file 1 [file healthcare-09-01000-s001.zip › healthcare-1305269-supplementary.pdf]

healthcare

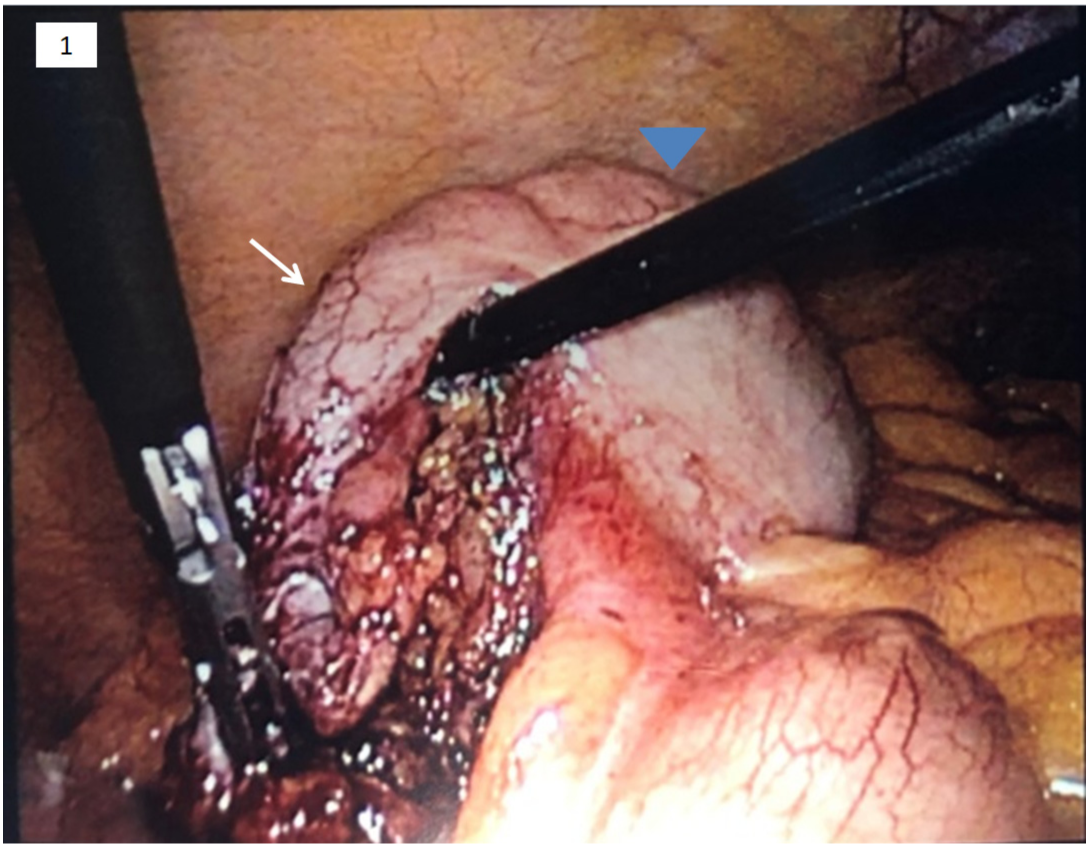

Figure S1. Enlarged appendix (arrow) and cecum (arrow head) were found during the procedure of laparoscopic appendectomy.

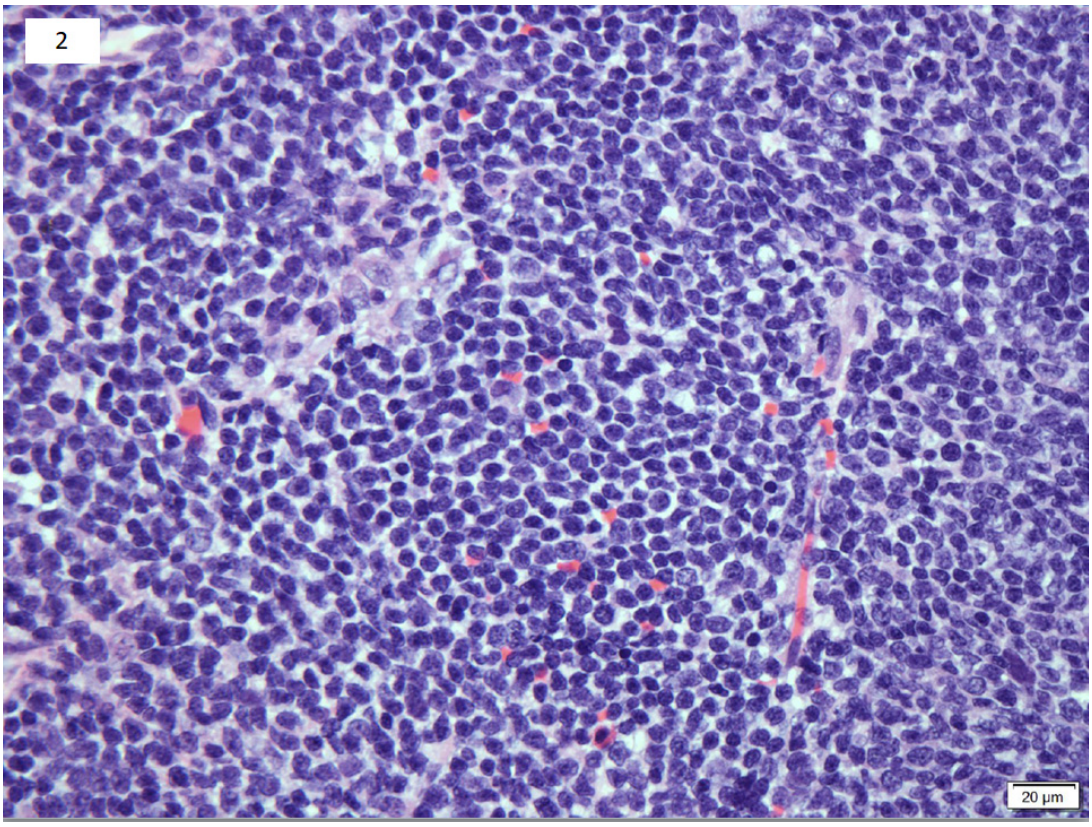

Figure S2. Under high-power photography of microscopy, tumor cells were characterized by small to medium-sized monotonous atypical lymphoid cells with irregular nuclear contours, condensed chromatin, and inconspicuous nucleoli.
